# Leishmaniasis in IBD Patients: Challenges of a Rare Opportunistic Disease

**DOI:** 10.1002/ueg2.12753

**Published:** 2025-01-08

**Authors:** Candida Abreu, Rafael Rocha

**Affiliations:** ^1^ Serviço de Doenças Infeciosas Hospital de São joão Unidade Local de Saúde de São João Porto Portugal; ^2^ Faculdade de Medicina Departamento de Medicina RISE‐Health Universidade do Porto Porto Portugal

*Gutierres* et al. [1] present an insightful retrospective observational study on clinical *Leishmania* infection (LI) in adult patients with inflammatory bowel disease (IBD) receiving immunomodulatory and/or immunosuppressive therapies. This multicenter study analyzed data from 73 IBD patients diagnosed with leishmaniasis between 2012 and 2022, across 26 hospitals in Spain. Notably, it is the largest study of its kind, and given the rarity of LI in IBD patients, the number of reported cases is remarkable.

Leishmaniasis is a neglected, opportunistic parasitic disease caused by a diverse group of protozoa of the genus *Leishmania*, transmitted by sand fly vectors. *Leishmania infantum* is the predominant species in the Mediterranean endemic regions, with humans serving as accidental hosts of the parasite, which primarily targets the mononuclear phagocyte system. *Leishmania* infection can range from an asymptomatic state to various clinical forms: cutaneous, mucosal, and/or visceral disease. Persistent asymptomatic infection, likely due to the absence of sterilizing immunity, can progress to symptomatic disease, particularly in the setting of impaired T‐cell immunity [2]. A Th1‐dependent cell‐mediated immune response, involving cytokines such as interferon‐gamma (IFN‐γ), tumor necrosis factor‐alpha (TNF‐α), and interleukin‐12 (IL‐12), is critical for controlling LI [3]. However, the mechanisms underlying disease progression and specific risk factors remain incompletely understood.

Historically, LI by *L. infantum* was predominantly diagnosed as visceral leishmaniasis (VL) in HIV‐infected patients with severe CD4 lymphocyte depletion, prior to the availability of highly effective antiretroviral therapy [4]. Similarly, LI is a rare but recognized complication in solid organ transplant recipients, where immunosuppressive therapies targeting T‐cell function predispose to infection [5].

In this study, the authors emphasize that nearly all IBD patients diagnosed with LI were receiving immunosuppressive therapy at the time of diagnosis, with 97% treated with anti‐TNF agents. Further studies are warranted to explore the risk profiles of newer biologics, such as IL‐12/23 or IL‐23 inhibitors, and small molecule therapies.

Localized cutaneous leishmaniasis (CL) was the most common presentation (82%), while VL occurred in 11% of cases, predominantly in older patients (mean age: 65 years). In contrast, in another study in rheumatic patients treated with anti‐TNF therapy [6], VL was reported more frequently than CL. These differences could be related to distinct patient profiles, as well as different dermotropism of local *Leishmania* strains.

The clinical course of localized CL was generally favorable, with 96% of cases achieving resolution within 12 months, irrespective of whether biological therapies were discontinued. This suggests that localized forms of LI may not always require the cessation of IBD therapies. However, a recent literature review [7] suggested caution, reporting refractory disease or progression to VL in cases in which immunosuppressive therapy was continued.

*Remaining questions* include whether screening for *Leishmania* infection in IBD patients from endemic areas prior to initiating immunosuppressive therapy would be beneficial. A study conducted in Catalonia, Spain [8], surveyed asymptomatic *Leishmania* infection in IBD patients receiving biological therapies. They identified a 10.9% positivity rate (via Western blot and/or qPCR); however, the short follow‐up duration precluded conclusions regarding the risk of reactivation. If screening is positive, the clinical implications remain uncertain—should biologic therapies be withheld or precluded? As such, in current practice, a reasonable approach seems to be no screening and regular clinical follow‐up for early detection of LI.

In this sense, the authors highlight the importance of maintaining a high index of suspicion for LI in patients with persistent cutaneous lesions, unexplained systemic symptoms (fever, organomegaly, cytopenia), or relevant epidemiological exposures, such as residence in or travel to endemic regions. In this cohort, the mean time to diagnosis was approximately 4 months, with some cases taking up to 2 years. CL, in particular, may be atypical in immunosuppressed patients, and misdiagnosed as eczema, psoriasis, or pyoderma gangrenosum. Advances in diagnostic methods for VL, such as molecular testing in blood [9], offer noninvasive alternatives to older techniques, such as bone marrow aspiration, improving the ability to detect cryptic infections.

Therapeutic considerations include the risk of relapse, which was historically high in HIV‐infected patients [4], but appears less frequent in IBD patients treated with biologics. The severity of immunosuppression likely plays a role, particularly when therapies are continued during treatment for LI. Conversely, the safety of reintroducing biologics following LI treatment remains a critical question. Analogous to tuberculosis reactivation [10], evidence suggests that reintroducing biologic therapy after adequate treatment for LI may be safe, but further data are needed.

While waiting for further research, greater awareness and improved diagnostic tools are essential to an early diagnosis and a better management of LI in immunosuppressed IBD patients.

## Conflicts of Interest

The authors declare no conflicts of interest.

## Data Availability

Data sharing is not applicable to this article as no new data were created or analyzed in this study.

## References

[1] L. Madero‐Velázquez , A. Mínguez , L. Mayorga , et al., and on behalf of Young Group of GETECCU , “Leishmaniasis in Patients With Inflammatory Bowel Disease: A National Multicenter Study of GETECCU,” United European Gastroenterology Journal. (n.d.) 10.1002/ueg2.12740 PMC12188349 39776301

[2] L. I. McCall , W. W. Zhang , and G. Matlashewski , “Determinants for the Development of Visceral Leishmaniasis Disease,” PLoS Pathogens 9, no. 1 (January 2013): e1003053, 10.1371/journal.ppat.1003053. Epub 2013 Jan 3. PMID: 23300451; PMCID: PMC3536654. 23300451 PMC3536654

[3] J. Alexander and F. Brombacher , “T Helper1/t Helper2 Cells and Resistance/Susceptibility to Leishmania Infection: Is This Paradigm Still Relevant?,” Frontiers in Immunology 3 (April 17, 2012): 80, 10.3389/fimmu.2012.00080. PMID: 22566961; PMCID: PMC3342373. 22566961 PMC3342373

[4] J. Alvar , C. Cañavate , B. Gutiérrez‐Solar , et al., “Leishmania and Human Immunodeficiency Virus Coinfection: The First 10 Years,” Clinical Microbiology Reviews 10, no. 2 (April 1997): 298–319, 10.1128/CMR.10.2.298. PMID: 9105756; PMCID: PMC172921. 9105756 PMC172921

[5] S. Antinori , A. Cascio , C. Parravicini , R. Bianchi , and M. Corbellino , “Leishmaniasis Among Organ Transplant Recipients,” Lancet Infectious Diseases 8, no. 3 (March 2008): 191–199, 10.1016/S1473-3099(08)70043-4. PMID: 18291340. 18291340

[6] L. S. Guedes‐Barbosa , I. Pereira da Costa , V. Fernandes , L. M. Henrique da Mota , I. de Menezes , and M. Aaron Scheinberg , “Leishmaniasis During Anti‐Tumor Necrosis Factor Therapy: Report of 4 Cases and Review of the Literature (Additional 28 Cases),” Seminars in Arthritis and Rheumatism 43, no. 2 (October 2013): 152–157, 10.1016/j.semarthrit.2013.01.006. Epub 2013 Jun 15. PMID: 23777708. 23777708

[7] L. Gimeno‐Pitarch , P. Almela , P. Nos , “Leishmania Infection in Patients With Inflammatory Bowel Disease: Case Series and Literature Review,” Gastroenterology and Hepatology 47, no. 1 (2024): 82–92, 10.1016/j.gastrohep.2023.04.001. English, Spanish. Epub 2023 Apr 13. PMID: 37061089. 37061089

[8] M. C. Guillén , M. M. Alcover , N. Borruel , et al., “ *Leishmania Infantum* Asymptomatic Infection in Inflammatory Bowel Disease Patients Under Anti‐TNF Therapy,” Heliyon 6, no. 5 (May 8, 2020): e03940, 10.1016/j.heliyon.2020.e03940. PMID: 32420499; PMCID: PMC7218013. 32420499 PMC7218013

[9] L. Galluzzi , M. Ceccarelli , A. Diotallevi , M. Menotta , and M. Magnani , “Real‐time PCR Applications for Diagnosis of Leishmaniasis,” Parasites & Vectors 11, no. 1 (May 2, 2018): 273, 10.1186/s13071-018-2859-8. PMID: 29716641; PMCID: PMC5930967. 29716641 PMC5930967

[10] T. T. Brehm , M. Reimann , N. Köhler , and C. Lange , “(Re‐)introduction of TNF Antagonists and JAK Inhibitors in Patients With Previous Tuberculosis: A Systematic Review,” Clinical Microbiology and Infections 30, no. 8 (August 2024): 989–998, 10.1016/j.cmi.2024.04.011. Epub 2024 Apr 23. PMID: 38663653. 38663653

